# Impacts of Thermal Environments on Health Risk: A Case Study of Harris County, Texas

**DOI:** 10.3390/ijerph18115531

**Published:** 2021-05-21

**Authors:** Bumseok Chun, Misun Hur, Jaewoong Won

**Affiliations:** 1 Urban Planning and Environmental Policy, Texas Southern University, Houston, TX 77004, USA; bum.chun@tsu.edu; 2 Department of Geography, Planning, and Environment, East Carolina University, Greenville, NC 27858, USA; hurmi@ecu.edu; 3 Department of Real Estate, Graduate School of Tourism, Kyung Hee University, Seoul 02447, Korea; 4 Department of Smart City Planning and Real Estate, Kyung Hee University, Seoul 02447, Korea

**Keywords:** health risk, thermal environment, green infrastructure, structural equation model, land use, urban environment

## Abstract

The loss of green spaces in urbanized areas has triggered a potential thermal risk in the urban environment. While the existing literature has investigated the direct relationship between urban temperatures and health risks, little is known about causal relationships among key components of urban sustainability and health risks, through a pathway involving urban temperature. This study examined the multiple connections between urbanized land use, urban greenery, urban temperatures and health risks in Harris County, Texas. The census tract-level health data from the 500 Cities Project (Centers for Disease Control and Prevention) is used for analysis. Structural equation model analyses showed that the urban temperature played a mediating role in associations between urbanized land use, urban greenery and health risk. Urban vegetation is associated with a decrease in health risks, while urban land use has associations with an increase in health risks. Findings suggest that proactive policies tailored to provide rich urban greenery in a neighborhood can alleviate urban land use effects on health risks.

## 1. Introduction

The United States Environmental Protection Agency (EPA) reports that a total of more than 11,000 people have died from heat-related causes in the past 40 years, which is between 0.5 and 2 deaths per million people [1]. When considering underlying and contributing causes of death, the death ratio has increased by more than threefold if we compare the 2010s to the 1980s [1]. However, unlike other disastrous natural events—e.g., hurricanes and tornadoes–heat-related events are not considered a disaster incident by the US Federal Emergency Management Agency (FEMA). With frequent extreme ‘killer heat’ days, Sherman (2020) alerts cities to the need to develop plans for extreme heat [2]. Planning Magazine also warned planners to be “heat-ready,” which is just as crucial as being storm-ready [3].

The United Nations forecasts that 70% of the world’s population will live in urban areas by 2050 [4]. Urbanized areas often experience higher temperatures than outlying areas, i.e., the urban heat island (UHI) [5]. The UHI further amplifies heat-related threats to city dwellers. Research has suggested the adopting vegetation and bodies of water through design and land use policies as solutions to address the UHI problem in cities [6,7,8]. Although such findings are helpful, their scope is limited to looking at the direct relationships between UHI and selective urban features. Thus, it is vital to investigate the complex interplay among different components to understand the connections among features more clearly. 

This paper takes a comprehensive approach by highlighting the interwoven associations among land use, urban greenery, temperature, and health risk using structural equation modeling (SEM). Below are the detailed research questions: 

Question 1: Which type of land use affects urban temperature, especially surface temperature?

Question 2: What type of greenery features (e.g., tree canopy cover, tree height, and glass cover) influences temperature? 

Question 3: Does the thermal environment adversely affect public health? 

Question 4: Are there associations between land use, urban greenery, temperature, demographics, and health risk? If so, in which direction? 

Harris County in Texas, where the Houston metropolitan area sits, experiences extreme heat, which can lead to severe environmental and health-related problems. According to the Heat Surveillance Monthly Report, heat-related illness and mortality events continued to increase, from 150 cases to 256 cases during the three-year period from 2013 to 2016 [9]. Taking Harris County as the study area, we sought to elucidate the underlying meaningful associations between urban land use, greenery characteristics, urban temperature, and residents’ health—i.e., chronic disease. 

## 2. Literature Review 

### 2.1. Land Use Pathway for Urban Temperature and Health

Studies have reported that built-up areas are critical contributors to increases in surface temperatures. Maloley (2009) analyzed land cover changes during the past two decades in Canada’s Toronto area and found a significant effect of new development on the increase in surface temperatures [10]. Other studies examined which land use types have the most influence on urban temperatures. Jusuf et al. (2007) examined the effects of land use on surface temperatures and found that commercial, residential, airport, and industrial land uses created higher temperatures than parks [11]. Rinner and Hussain (2011) analyzed various land uses (e.g., industrial, institutional, residential, open area, parks and recreational spaces, and water bodies) related to the Toronto area’s surface temperature and found that surface temperatures were higher for commercial and industrial land use, characterized by a high proportion of built-up surfaces [12]. At the same time, parks and recreational spaces had lower surface temperatures.

Health studies have found significant evidence showing close associations between land use patterns and community health promotion [13,14]. These studies take a similar perspective to that of the recent Smart Growth movement in urban planning. Compact and diverse land use choices create more accessible destinations that encourage walking trips as a way to promote a healthy lifestyle [15]. A modal shift from private vehicles to walking and public transit has been encouraged by central planning policies. However, the history of modern urban development has shown high-density land use and built environments being the leading causes of rising urban temperatures. Research has revealed that urban heat can increase mortality risks such as respiratory illness, heart disease, and cardiovascular disease [16]. However, the link between land use and health risk through urban temperature pathways is not yet clear. This study examines the associations between various land uses and health risks, through urban temperature as the mediator. 

### 2.2. Urban Greenery Pathway for Urban Temperature and Health

Among various land uses, green spaces—i.e., parks and open spaces—have received attention in the literature due to the various potential benefits they can bring. Empirical research has found evidence that green spaces improve urban environmental quality—including air, noise, and temperature [17]. Green spaces also boost residents’ psychophysiological well-being through their therapeutic and stress-relieving effects [6]. Greenery promotes healthy behaviors (i.e., walking in a neighborhood) [18], which foster substantial social capital as an opportunity for walking and social interactions [19]. Research confirms the benefits of strong social capital for psychological resiliency [20]. Gill et al. (2007) highlighted the critical role of green infrastructure as a vital adaptation strategy for climate change in urban environments [21].

Such green benefits including social, psychological, and environmental aspects have positively influenced community health. The literature suggests that residents living in a community with an abundance of greenery tend to have better health (e.g., less morbidity, less mortality) and well-being (e.g., happiness) [22]. They benefit from clean air, more opportunities for physical activities, recovery from mental stress, and social interaction [23,24]. Residents in a greener neighborhood had higher satisfaction with their environment, which positively relates to mental health [25] and physical health [26]. Research reported that people in green environments—compared to those who are not—have lower blood-pressure-related problems [6]. They are also less overweight/obese [27], have lower morbidity and mortality ratios [19], and have fewer cardiovascular disease cases [28].

Despite the numerous benefits of urban greenery, it is unclear which green features contribute to urban temperature and health. The greenery studied is often limited to an urban park or open space [29]. Although the value of parks and open spaces is immense, other greenery features, such as street trees and grass in a neighborhood, could bring more or less direct and significant benefits to residents. Greenery such as street trees, plants, and grass alongside or on sidewalks may have significant potential to support planning strategies for establishing more relaxed environments. At the same time, they are still effective contributors to creating walkable and healthy neighborhoods. Our study takes various greenery features into consideration and searches for specific urban greenery features that alleviate urban temperatures and improve residents’ health. 

### 2.3. Multiple Pathways among Land Uses, Green Spaces, Urban Temperature and Health Risk

The outdoor temperature (high or low) affects health directly and indirectly. High urban temperatures, predominantly during heat waves, adversely influence health. Heatwaves intrude on nervous system functions and can lead to the termination of vital rhythmic activity as they obstruct the balance of nerve signals with abnormal electrolyte levels [30]. Heat exposure can also increase chronic disease risks—e.g., obesity, high blood pressure, stroke, and asthma [31]. A chemical reaction in the atmosphere assisted by hot temperatures worsens air quality, and this may cause outdoor discomfort relevant to asthma [32]. High temperatures can hinder outdoor physical activity [33] and might adversely impact the body’s metabolism rate. Edwards et al. (2015) also confirmed reductions in physical activities for every ten additional degrees of heat in young children in their longitudinal cohort study [34]. A lack of such physical exercise might cause obesity and high blood pressure [35]. While these reports have addressed the importance of thermal environments from the community health viewpoint, empirical evidence needs to be provided.

In this study, we examine whether and how land use and green space are associated with health risk through the pathway of urban temperature. The interwoven associations among urban environmental aspects and health are thoroughly investigated.

## 3. Study Area

**Figure 1:**
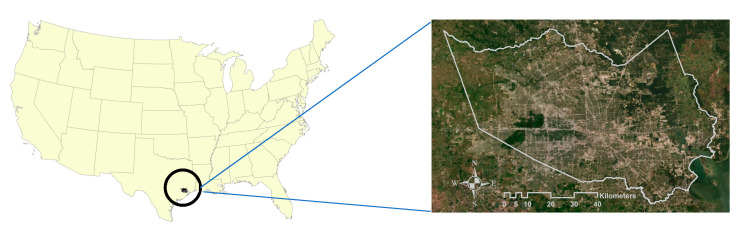
Location of Harris County in Texas.

This study focused on Harris County (area: approximately 4602.41 km^2^), Texas, where Houston sits. Figure 1 shows the geographic location of Harris County. With over two million people as of 2010, Houston is the most populated city in Texas. The U.S. Census’s estimated population of Houston (approx. 2.3 million) ranks it as the fourth-largest urban area in the United States, after New York City (approx. 8.3 million), Los Angeles (approx. 4.0 million) and Chicago (2.7 million) [36]. The Houston-The Woodlands-Sugar Land Metropolitan Statistical Area has grown significantly in recent decades from 1 million residents in 1950 to 3.3 million in 1980 [37] and 7.1 million in 2019, with increased built-up urban areas [36].

**Figure 2:**
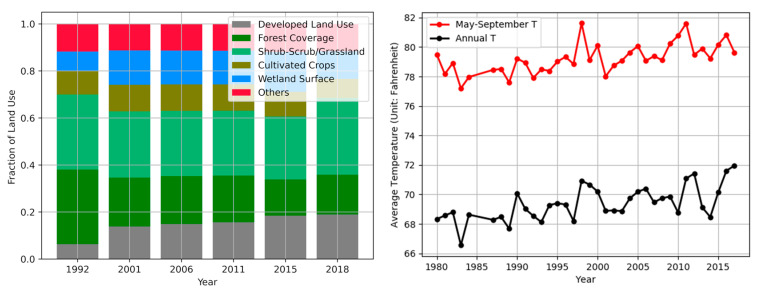
Land use and annual temperature variations in Harris County.

Figure 2 shows the longitudinal trend of land use compositions (left) and temperature (right) (data resources: the U.S. Geological Survey (USGS) and the Houston-Galveston Area Council). The left chart shows the increase in developed land uses defined by the National Land Cover Data (NLCD) while it shows the decrease in forest coverage over time in Harris County. The graph on the right shows the increased temperatures during the same period. Hot temperatures make outdoor activities more difficult, exacerbating discomfort.

In terms of public health, chronic diseases are increasing and are the leading causes of death in Harris County and in the nation [9]. In particular, the rates of obesity and overweight have risen rapidly since the late 1980s, with a record high of 65% of people being obese in Harris County in 2013. National rates of the same conditions have increased from 56% to 70% over the same period, elevating the potential risk of developing other health problems, such as heart-related disease, hypertension, arthritis, kidney disease, stroke, asthma, and infertility [38]. Not only the obesity rate but other disease rates such as chronic respiratory diseases (32%) and high blood pressure (32.4%) are also higher in Harris County, Texas, than U.S. averages of 7.7% for chronic respiratory and 32% for high blood pressure in 2016. These facts suggest that this study area is suitable for analyzing the thermal environment’s effects on health risk. 

## 4. Methodology

### 4.1. Data Sources, Factors and Variables 

Four chronic diseases—obesity, high blood pressure, stroke, and asthma— were used as the primary endogenous variables for the Health Risk factor. We obtained these data from the 500 Cities Project sponsored by the Robert Wood Johnson Foundation and Centers for Disease Control and Prevention (CDC) in 2015 (https://www.cdc.gov/500cities/index.htm (accessed on 23 April 2021)). The 500 Cities Project initially used publicly available data on 27 types of chronic diseases at the city and census tract levels. Each variable ranged from 0% to 100% of the population for each disease per census tract. Our research employed chronic disease data from the 634 census tracts in Harris County, omitting the 152 census tracts with no data reported.

For the Land Use factor, we adopted the parcel data from the Harris County Appraisal District (HCAD). This included ten categories of land use type: single-family residential, multi-family residential, commercial, office, public/institutional, industrial, transportation/utility, park/open spaces, undeveloped, and agricultural uses. However, we re-grouped them into five land use patterns based on similar environmental characteristics to reduce statistical bias caused by the high degree of similarity between land uses. Then, we calculated the percentage coverage by each category from all available land uses per each census tract.

For the Urban Greenery factor, we applied an advanced geospatial analysis. Light Detection and Ranging (LiDAR) was used for vegetation height, and high-resolution Color-infrared (CIR) aerial photography data was used for the density of green space. Proxy tree heights generated from the LiDAR were applied to cells with Normalized Differential Vegetation Index (NDVI) value greater than 0.2, representing green space [39]. We estimated vegetation densities with the average tree height of each census tract. Heights greater than 3.05 m (≈10 feet) were treated as trees (canopy), and others were considered as grasslands covering all types of vegetation, such as grassland/herbaceous, shrub/scrub, and pasture/hay [40]. We used the 2008 LiDAR and the 2014 CIR image data produced by the Houston-Galveston Area Council (H-GAC). The CIR images were provided with a 3-m horizontal resolution, which can capture tree canopies in parks and along the streets. Although there were discrepancies in data sources due to data availability issues, it did not create any data construction problems because there has been little construction and demolition in the study area. 

Thermal environments, directly and indirectly, affect human comfort and health in terms of heat-related mortality and morbidity. We measured two temperature variables—average Daytime Land Surface Temperature (DLST) and average Nighttime Land Surface Temperature (NLST)—using the MODIS (Moderate Resolution Imaging Spectroradiometer) MOD11A2 product at 1-km spatial resolution (https://earthdata.nasa.gov/ (accessed on 10 January 2019)). Satellite-derived temperatures can be a way to remove uncertainty regarding missing information from weather monitoring stations. In this study, we employed the average DLST and NLST measured in 2014. 

Various demographic characteristics were also considered. A meta-analysis conducted by Romero-Lankao et al. (2012) identified diverse socio-demographic variables supporting the association between temperature and heat-related diseases [41]. Of these, we selected three that could avoid multicollinearity between variables. First, a different racial distribution could cause different exposure levels to health risk because of different physical, mental, and social activity. Second, education level is also a key factor to determine potential exposure to health risk because it affects adaptive functional capability in terms of a healthy lifestyle. Last, the elderly has been classified as a vulnerable population, implying that they could be affected by the thermal environment. All demographic data were retrieved from the U.S. Census (2014 American Community Survey 5-year estimates) [42]. 

### 4.2. Statistical Analysis 

We used the SEM analysis with Covariance Analysis and Linear Structural Equations (CALIS), using the maximum likelihood method of parameter estimation with SAS 9.4. The SEM is a hybrid model with a two-step process: first, a measurement model and then a structural path model [43]. The measurement model was used to describe the relationships between the latent factors and their indicator variables. A confirmatory factor analysis (CFA) was used for the procedure. Three goodness-of-fit indexes determined the model fit with the variables and factors and their interwoven associations. For more information, see Table 2. We used five factors with 18 variables (Table 1) and the variance–covariance matrix (*n* = 634). Among the factors, land use, urban greenery, and demographics were exogenous factors, while temperature and health risk were endogenous factors in the suggested SEM model. 

## 5. Findings

### 5.1. Descriptive Statistical and Comparative Spatial Analyses 

The descriptive analyses using the means and the spatial distributions of each variable enabled us to examine Harris County’s circumstances in 2014 at the census tract level. A summary of all factors and variables used in this research can be found in Table 1, with simple descriptive statistics, units, and descriptions. 

First, the percentage of the population with obesity or high blood pressure was significantly higher than those with asthma or stroke. Second, the majority of Harris County land use in 2014 was for residential use, followed by commercial and other land use (likely to be green-related land use). Third, the mean value of the grass cover was close to double that of tree cover. As for demographic characteristics, compared to national averages, the county had more non-white, highly educated, and older population (compared to 26.2% non-whites, 17.2% for bachelor or higher education attainment, and 13.7% people 65+ age-old, nationally).

**Figure 3:**
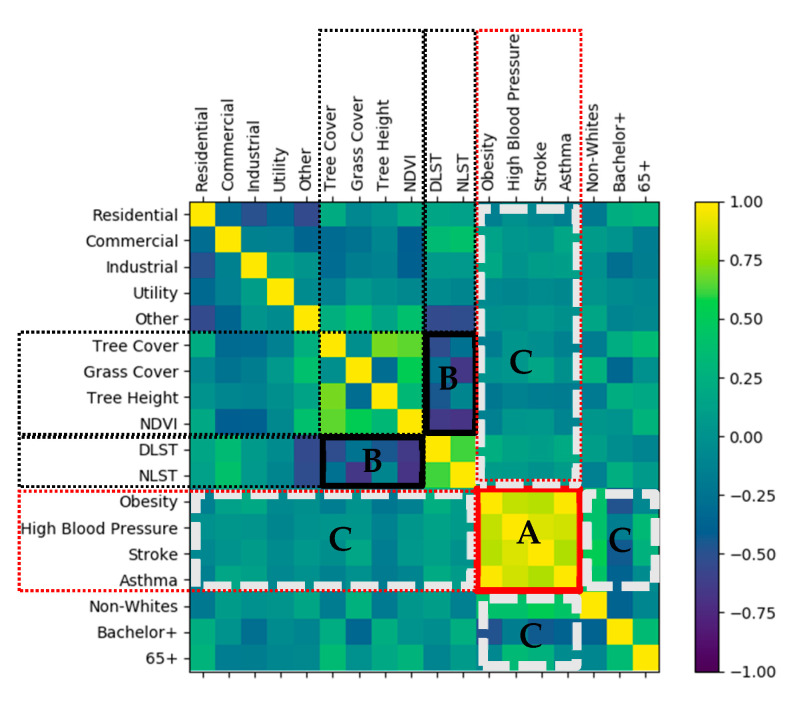
Correlation Heatmap.

Figure 3 visualizes the correlation matrix of variables as a heatmap. The map helps to identify the incidence patterns as well as the anomalies among the variables. Yellow means positive, and blue means negative. The stronger the color, the larger the correlation magnitude. One outstanding finding was that all health risk variables turned out to be positively correlated to each other (box A in red). In contrast, there seemed to be negative correlations between temperature variables and urban greenery variables (boxes B in black). However, the strengths of the associations of health risks with land use, urban greenery, temperature, and most demographic variables (boxes C in white) were unclear.

**Figure 4:**
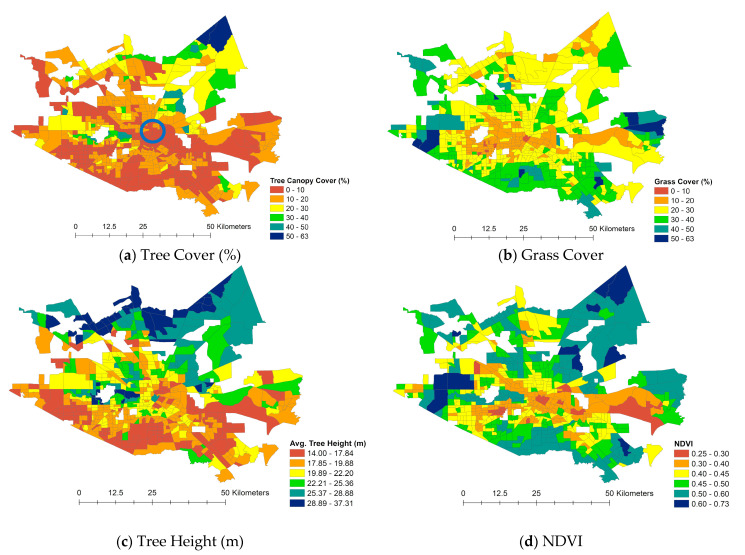
Spatial Patterns of Urban Greenery (Note: blue circle locates downtown Houston).

To explore each factor’s local patterns, we further looked at the spatial distributions of the variables. Figure 4 illustrates the spatial patterns of each urban greenery variable at the census tract level. First, the NDVI—representing the vegetation coverage—had similar spatial distributions to grass cover. The central east–west band with lower values for both features is the Buffalo Bayou, a slow-moving river that flows through Houston. Second, the spatial patterns of tree cover and tree height were similar, with the whole span of the southern edge of the study area having lower values, while the northeastern areas had higher values. There was approximately 13.3% more grass coverage than tree coverage in the study area. 

**Figure 5:**
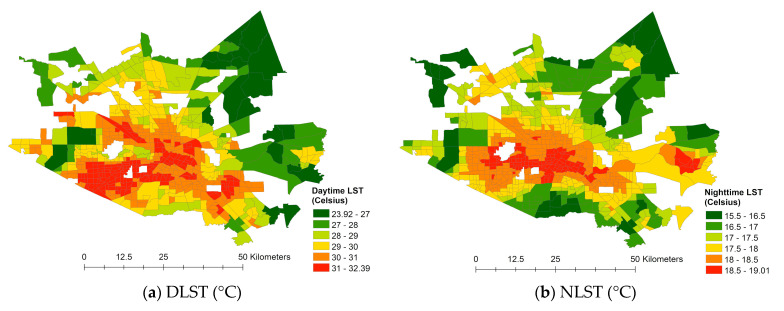
Spatial patterns of temperature.

Figure 5 presents DLST and NLST. In 2014, the average DLST was 30.01 °C, and the NLST was 17.83 °C in Harris County. Expectedly, the urban core temperature—for the downtown Houston area—remained higher than the surroundings, even at nighttime. For example, the urban core temperature was 8.37 °C higher in the daytime and 3.34 °C higher in the nighttime than its surroundings.

**Figure 6:**
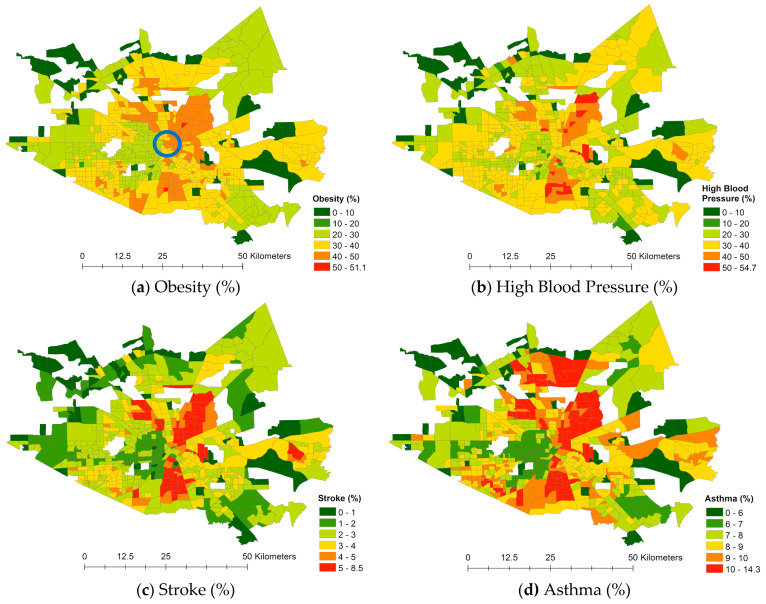
Spatial patterns of health risk (Note: Blue circle locates downtown Houston).

Figure 6 maps the percentage of the population with each health risk variable per census tract. Relatively high health risk areas are commonly observed in the northeast, northwest, and southern directions from downtown Houston. The census tracts in both orange and red for obesity and high blood pressure indicate that more than 40% of the population had obesity and hypertension symptoms during the research year, which is a very high percentage and indicates significant public health problems.

### 5.2. Measurement Model

Table 2 lists the Goodness of Fit Indexes of three models—our initial theoretical model, the measurement model, and the SEM model. Among various fit indexes, we used the Goodness of Fit Index (GFI), the Bentler Comparative Fit Index (CFI), and the Bentler-Bonett Normed Fit Index (NFI). With the lower risk of producing biased estimates in small samples [44], researchers often use these indexes. Values over 0.9 on its index indicate an acceptable fit. Since the initial theoretical model had a poor fit, we revised the model. Hatcher (1994) suggests reassigning or altogether dropping an indicator from a model rather than assigning it to two factors simultaneously (maintaining the unifactorial characteristics of each indicator variable) [45]. After a series of modifications using the CFA analysis, we found a model in which all fit indexes fell into a reasonable error of approximation (Table 2). Therefore, the model was tentatively accepted as the study’s final measurement model, and several tests were conducted to assess its reliability and validity (Table 3). 

The measurement model resulted in only nine indicator variables being significant among the suggested 18 variables. Among the land use variables, only commercial and utility were significant; for urban greenery, variables related to trees were significant; and for health risk, all variables except asthma were found to be significant. The model indicated that both the DLST and NLST variables were significant. However, none of the demographic variables were validated in the associations with other latent variables. As a result, we excluded all invalid variables and the demographic factor. 

Table 3 shows the measurement model’s factors and indicators with the results from the reliability and validity assessments. The Standard Factor Loadings for each variable are presented in the second column of the table. The health risk, urban greenery, and temperature factors showed higher than 70 Composite Reliability, which reflects the internal consistency of the indicators measuring the given factor [46]. A Composite Reliability value of 70 was considered as being the minimum acceptable level of reliability for the instruments. Hatcher (1994) suggested the use of Variance Extracted Estimates to “assess the amount of variance that is captured by an underlying factor in relation to the amount of variance due to measurement error (p. 331)” [45]. Similar to reliability assessments, all factors except land use reported higher than the acceptable level of 50. The findings could be interpreted such that, for example, 85% of the variance was captured by the Health Risk factor, and only 15% (1 − 0.85 = 0.15) was due to measurement error. As explained, most reliability and validity test findings generally supported the factors and their indicators. Although both the reliability and the variance extracted estimates did not advocate land use and its indicator variables, both variables’ Convergent Validity (t-scores) were significant at *p* < 0.001. This confirmed that the indicator variables—commercial and utility—effectively measured the same construct—land use factor [43]. Therefore, the measurement model was taken to proceed further in the structural path model estimations. 

### 5.3. Structural Path Model (SEM Model)

**Figure 7:**
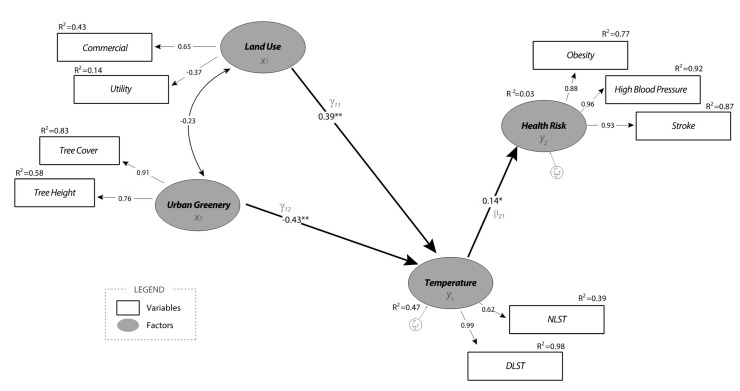
The Final SEM Model. (Note: *: significant at 95% confidence level, **: significant at 99% confidence level).

Figure 7 shows the final SEM model with the estimated coefficients among all factors and variables suggested by the measurement model. In the SEM model, we visualize factors using ovals, and the variables have rectangular shapes. The exogenous factors are on the left, while the endogenous factors are on the right to show the associations/effects naturally flowing from left to right on the model. The R-squared values are also provided outside of every endogenous variable to explain how much each endogenous variable contributes to supporting a latent variable. The single-headed straight arrows represent the associations’ directional strength, while the double-headed curved arrows show the reciprocal correlations. 

The structural model shows the direct and indirect associations among factors. A direct association appears as an arrow between temperature and health risk factors, between land use and temperature factors, and between urban greenery and temperature factors. Readers can also follow the indirect associations between factors through a mediating factor. For example, health risk and land use factors are only associated indirectly via temperature as the mediator. Similarly, an indirect association between health risk and urban greenery can be found via temperature as the mediator. The direct association of health risk with temperature had a positive effect of moderate strength (0.14). The findings show that people who lived in an area with relatively higher nighttime and daytime temperatures—a thermal environment—also showed higher incidences of obesity, high blood pressure, and stroke. Outdoor activity relating to thermal discomfort could significantly contribute to the increasing prevalence of these health risks. All estimated parameters, including direct, indirect, and total effects, are summarized in Table 4. 

As mentioned above, both land use and urban greenery had indirect associations with health risk through the mediating factor of temperature. The findings suggest that land use indeed has a positive association with health risks. In particular, the area with higher proportions of commercial and industrial land use reported higher daytime and nighttime temperatures with a large effect size of 0.39 and higher incidences of health risks with a small effect size of 0.05 (indirect effect calculated by multiplying 0.39 and 0.14). On the other hand, urban greenery has negative associations with temperature and health risks. Areas with higher tree canopy coverage and taller (mature) trees show significantly lower temperatures throughout the day. The effect size between urban greenery and temperature was the greatest (−0.43) among all associations in the model. The findings further contribute to the relationship between urban greenery and health risk—the indirect association between these factors was −0.06 (calculated by −0.43 × 0.14). It is important to note that these relations could never be disclosed using traditional linear regression analysis because none of the mediating variables would be included. The advanced SEM analysis handled the tasks perfectly, which is an additional contribution of this research to current understanding in the field.

Lastly, the SEM model also showed a negative correlation (−0.23) between two exogenous factors—land use and urban greenery. This link indicates that areas with commercial and utility land uses often had a lower degree of tree canopy coverage and relatively smaller trees in terms of height. For example, the vast majority of ground surfaces used for commercial land use were covered by impervious pavements near parking lots, roadways, and buildings, thereby reducing green spaces. In general, the implementation of utility land use for transportation and conventional utility also reduced vegetation. 

## 6. Discussion 

Using publicly available secondary databases, we conducted a series of advanced GIS analyses by applying remote sensing techniques. Using descriptive statistical and spatial distribution analyses, we explained the trends of all variables to provide a general understanding of the study area. All variables were then spatially joined together at the census tract level to run an advanced statistical analysis. The SEM results revealed interwoven relationships among land use, urban greenery, temperature, and health risk factors.

This research’s most significant finding is the vital role of the temperature factor as the mediator between both land use and urban greenery and health risks. The results show that areas with higher commercial and industrial land use ratios with less tree canopy and smaller trees were more likely to have relatively higher land surface temperatures throughout the day. The associations further contribute to residents developing higher incidences of obesity, high blood pressure, and stroke. Additional highlights can be stated. Among various land use types, only commercial and utility uses turned out to be significant types that contributed to increasing the land surface temperature regardless of the time of day. Such land use types intensify the UHI effect through vast open grounds—e.g., parking lots—often covered by impervious concrete surfaces. Air conditioning in commercial buildings also differs dramatically from cooling in homes. Commercial buildings are generally larger, use much energy, and ventilate a significant volume of heat. Hence, their urban temperature impacts could be detrimental, considering the universal prevalence of such land uses in the urban core. 

This research further contributes to the literature by looking at different greenery features separately—i.e., tree canopy cover, tree height, grass cover, and the NDVI. Although grass coverage seems to be a dominant feature that determines the NDVI more than trees, only tree cover (canopy) and height (size) were significant contributors to Harris County’s land surface temperatures. Considering the positive effects of greenery on environmental and health benefits, we suggest that proactive urban policies and additional actions to plant more tall and leafy trees are pursued. Urban neighborhoods filled with abundant green features with various tree types, maturities, and coverages would further promote environmental sustainability as a related benefit, which could lead to a healthy community. Another strength of this research is the broad applicability of its methods. Since we used publicly available data searching for factors that contribute to public health, researchers can apply the techniques in other areas. Advances in SEM analysis would add strength to the research. Indirect relationships among factors cannot be projected using traditional linear regression analysis. The other strength is the geographical unit of analysis. Previous health literature often utilized aggregated information on a geographically large scale due to the issue of privacy [47,48]. Therefore, their findings are difficult to suggest local health implications such as spatial patterns of health outcomes. In our research, we presented the potentials of using census tract-level detailed data from the CDC. Since the findings are specific to the environmental characteristics of Harris County, the implications are also explicitly related to local health needs. 

The research limitations could highlight directions for future research. First, interestingly, none of the demographic characteristics was associated with any other factor in this study. Perhaps the choice of demographic measures did not fit, or unknown variables prevented us from understanding the associations between the demographic factor and all other factors. Second, we did not take the temperature threshold or heat durations into consideration. Since the study area is one of the hottest cities throughout the year in the USA, we suspect that the temperature threshold and heat durations could influence the residents’ health conditions in various ways. Third, our study is empirical research with a specific interest in Harris County, TX. The model could be applicable in other cities with similar characteristics–megacities. Future research with comparisons could be advantageous. Lastly, we only used the LST as a representative variable of urban temperature associated with health. However, we understand that many other meteorological variables can be just as crucial to human health. With the increase in urban temperatures, changes in other variables such as humidity, barometric pressure, precipitation, and UV radiation can occur. Schneider and Breitner (2016) stated that the interplay of temperature with air pollution is also critical. Future research to address these limitations is needed [49].

## 7. Conclusions

This study used satellite image and census tract-level health data to examine complex associations among land use, urban greenery, surface temperatures, and health risks. This study found that the urban land uses covered mainly by concrete surfaces of building contributed to health risks whereas urban greenery such as trees along the streets and between buildings were significant for decrease in health risks. The findings of this research align with recent innovative initiatives, including the Sustainable Sites Initiative (SSI, http://www.sustainablesites.org/ (accessed on 23 April 2021)), Leadership in Energy and Environmental Design for Neighborhood Development (LEED-ND, http://leed.usgbc.org/nd.html (accessed on 23 April 2021)), Enterprise Green Communities Criteria (https://www.enterprisecommunity.org/solutions-and-innovation/green-communities/criteria (accessed on 23 April 2021)), and the Healthy Development Measurement Tool (HDMT, www.thehdmt.org (accessed on 23 April 2021)). With the tremendous efforts that have been made, understanding of the adverse impacts of UHIs have grown substantially, and it seems this trend will continue in the future. For a more sustainable, well-connected, and healthy neighborhood, continuing efforts to address this issue’s various aspects are needed.

## Figures and Tables

**Table 1:** Factors, Variables, and Descriptive Statistics.

Factor	Variable	Mean	SD	Min	Max	Description
Health Risk	Obesity	30.93	11.48	0	51.1	The population with obesity (%)
	High Blood Pressure	29.68	11.08	0	54.7	The population with high blood pressure (%)
	Stroke	2.82	1.59	0	8.5	The population with stroke (%)
	Asthma	7.87	2.71	0	14.3	The population with asthma (%)
Land Use	Residential	55.76	17.54	0	98.76	Single-family and multi-family residential land use (%)
	Commercial	16.54	11.80	0	100	Commercial, office, and public/institutional land use (%)
	Industrial	7.09	9.19	0	65.77	Industrial land use (%)
	Utility	2.69	6.72	0	97.53	Transportation and utility land use (%)
	Other	17.88	13.67	0	79.76	Other land use, i.e., parks, open space, agricultural land, undeveloped and unclassified land use (%)
Urban Greenery	Tree Cover	13.33	8.88	0	0.626	Tree canopy cover (%)
	Grass Cover	26.59	9.67	4.32	59.75	Grassland cover (%)
	Tree Height	21.01	4.07	14.8	37.3	Average tree heights (m)
	NDVI	0.45	0.08	0.25	0.24	Annual average vegetation coverage based on land surface reflection of satellite images (no unit: 0–1 range)
Temperature	DLST	30.01	1.38	23.92	32.29	Average annual daytime land surface temperature (°C)
	NLST	17.83	0.65	15.67	19.01	Average annual nighttime land surface temperature (°C)
Demographics	Non-Whites	39.37	22.27	0	54.40	Non-whites (%)
	Bachelor+	18.87	17.60	0.30	67.68	People with bachelor’s or higher degree (%)
	65+	9.64	5.18	0	30.50	People 65+ years old (%)

**Table 2:** The goodness of fit indexes of three models.

Model	N	GFI	CFI	NFI
Initial theoretical model	634	0.58	0.67	0.66
Measurement model	634	0.90	0.91	0.90
SEM model	634	0.90	0.91	0.90

**Table 3:** Factors and measures of the measurement model.

Factors and Measurements	Standardized Factor Loading	Convergent Validity (*t*) ^a^	Reliability	Variance Extracted Estimate
Health Risk			0.946 ^b^	0.854
Obesity	0.88	84.96	0.774	
High Blood Pressure	0.96	149.60	0.922	
Stroke	0.93	122.10	0.865	
Land use			0.056 ^b^	0.286
Commercial	0.66	9.60	0.436	
Utility	−0.37	−7.42	0.137	
Urban Greenery			0.831 ^b^	0.714
Tree Cover	0.93	34.00	0.865	
Tree Height	0.75	27.07	0.563	
Temperature			0.801 ^b^	0.679
DLST	0.98	30.74	0.960	
NLST	0.63	20.03	0.397	

^a^ All *t*-tests were significant at *p* < 0.001. ^b^ Denotes composite reliability.

**Table 4:** Estimated parameters in the final SEM model.

From Factor	To Factor	Direct Association	Indirect Association	Total Association
Land use	Temperature	0.39		0.39
Urban Greenery	Temperature	−0.43		−0.43
Land Use	Health Risk		0.05	0.05
Urban Greenery	Health Risk		−0.06	−0.06
Temperature	Health Risk	0.14		0.14
